# Preserving prion strain identity upon replication of prions in vitro using recombinant prion protein

**DOI:** 10.1186/s40478-018-0597-y

**Published:** 2018-09-12

**Authors:** Natallia Makarava, Regina Savtchenko, Peter Lasch, Michael Beekes, Ilia V. Baskakov

**Affiliations:** 1 Center for Biomedical Engineering and Technology, University of Maryland School of Medicine, 111 S. Penn St, Baltimore, MD 21201 USA; 2 Department of Anatomy and Neurobiology, University of Maryland School of Medicine, Baltimore, MD USA; 3 Centre for Biological Threats and Special Pathogens, Robert Koch-Institute, 13353 Berlin, Germany

**Keywords:** Prions, Prion diseases, Prion strain, Replication cofactors, Recombinant prion protein

## Abstract

Last decade witnessed an enormous progress in generating authentic infectious prions or PrP^Sc^ in vitro using recombinant prion protein (rPrP). Previous work established that rPrP that lacks posttranslational modification is able to support replication of highly infectious PrP^Sc^ with assistance of cofactors of polyanionic nature and/or lipids. Unexpectedly, previous studies also revealed that seeding of rPrP by brain-derived PrP^Sc^ gave rise to new prion strains with new disease phenotypes documenting loss of a strain identity upon replication in rPrP substrate. Up to now, it remains unclear whether prion strain identity can be preserved upon replication in rPrP. The current study reports that faithful replication of hamster strain SSLOW could be achieved in vitro using rPrP as a substrate. We found that a mixture of phosphatidylethanolamine (PE) and synthetic nucleic acid polyA was sufficient for stable replication of hamster brain-derived SSLOW PrP^Sc^ in serial Protein Misfolding Cyclic Amplification (sPMCA) that uses hamster rPrP as a substrate. The disease phenotype generated in hamsters upon transmission of recombinant PrP^Sc^ produced in vitro was strikingly similar to the original SSLOW diseases phenotype with respect to the incubation time to disease, as well as clinical, neuropathological and biochemical features. Infrared microspectroscopy (IR-MSP) indicated that PrP^Sc^ produced in animals upon transmission of recombinant PrP^Sc^ is structurally similar if not identical to the original SSLOW PrP^Sc^. The current study is the first to demonstrate that rPrP can support replication of brain-derived PrP^Sc^ while preserving its strain identity. In addition, the current work is the first to document that successful propagation of a hamster strain could be achieved in vitro using hamster rPrP.

**Electronic supplementary material:**

The online version of this article (10.1186/s40478-018-0597-y) contains supplementary material, which is available to authorized users.

## Introduction

Prion diseases or transmissible spongiform encephalopathies represent a class of lethal, transmissible neurodegenerative disorders of humans and animals [53]. The key event underlying prion diseases involves the conformational change of the α-helical, native, cellular form of the prion protein (PrP^C^) expressed by a host on a cell surface into a self-replicating, β-sheet rich, transmissible form (PrP^Sc^) [52]. PrP^C^ is posttranslationally modified with a glycophosphatidylinositol (GPI) anchor and up to two N-linked glycans; these modifications are carried over upon conversion of PrP^C^ into PrP^Sc^ [58, 59, 62]. Prions spread between organisms or from cell to cell by recruiting host-encoded PrP^C^ and replicating their disease-specific misfolded structures via a template-assisted mechanism [14]. According to this mechanism, PrP^Sc^ template recruits PrP^C^ expressed by a host and converts them into a new PrP^Sc^ with the folding pattern faithfully repeating that of the PrP^Sc^ template [14]. While prions can propagate indefinitely via serial passaging in wild type hosts or cultured cells, generating infectious prions in vitro de novo from recombinant PrP (rPrP) has been a challenge [5].

In the absence of cellular cofactors, rPrP readily adopts self-propagating β-sheet rich states including amyloid fibrils [7, 8]. While rPrP amyloid fibrils propagate well in vitro [9], they display miniscule specific infectivity in animals, as they do not recruit PrP^C^ effectively [15, 29, 39, 43]. When inoculated into wild type hosts, rPrP amyloid fibrils initiate a process of synthetic strain evolution that eventually lead to emergence of authentic PrP^Sc^ and clinical prion disease upon serial passaging [43–46]. Evolution of synthetic strains in vivo involves a transformation from the PrP folding patterns specific to rPrP amyloid fibrils, which do not accommodate well PrP with posttranslational modifications, to the folding pattern specific to PrP^Sc^ that can effectively recruit PrP^C^ with GPI anchor and N-linked glycans [42].

In the past decade, several studies demonstrated that authentic PrP^Sc^ infectious to wild type host could be generated using rPrP in vitro with assistance of cofactors of polyanionic nature and/or lipids using serial protein misfolding cyclic amplification (sPMCA) [21, 22, 63, 67]. Ma and colleagues showed that mouse recombinant PrP^Sc^, infectious to wild type mice, could be produced in sPMCA de novo using rPrP in the presence of the anionic phospholipid 1-palmitoyl-2-oleolyl-*sn*-glycero-3-phospho(1′-*rac*-glycerol) and total liver RNA [63, 67]. Supattapone and colleagues showed that phosphatidylethanolamine (PE) can be used as a sole cofactor for generating recombinant mouse PrP^Sc^ with high infectivity titers in sPMCA reactions seeded with mouse prion strains [21, 22]. Castilla and colleagues reported generation of several new strains in bank voles upon transmission of sPMCA-derived materials produced with assistance of several cofactors of polyanionic nature and bank vole rPrP as a substrate [23]. These studies established that highly infectious recombinant PrP^Sc^ could be generated from rPrP mixed with non-PrP components. In addition, these studies provided illustration that presence of different cofactors gave rise to different synthetic prion strains. However, it still remains unclear whether strain-specific features could be imposed on rPrP by brain-derived PrP^Sc^ seeds and, if so, can be faithfully replicated by rPrP, i.e. whether prion strain identity can be preserved by rPrP [60].

It has been well established that molecular features responsible for prion strain identity are well preserved when crude brain homogenates containing PrP^C^ is used for replicating prion strains in sPMCA [12, 28]. However, this is not the case, when rPrP is used for in vitro conversion. Studies using quaking-induced conversion assay revealed lack of infectivity and strain identity in rPrP conversion products generated by seeding of rPrP with a diverse range of prion strains originating from a number of species [65]. Surprisingly, supplementing PE as a sole cofactor to mouse rPrP conversion assays restored high titer of prion infectivity, yet did not preserve strain identity [22]. In fact, seeding of rPrP and PE mixtures with three mouse strains gave rise to recombinant PrP^Sc^ that produced a new strain in wild type mice with the same diseases phenotype regardless of the original strain used for the seeding [22]. Moreover, similar results were obtained using hamster PrP^C^ purified from hamster brains as a substrate and synthetic polyA as a sole cofactor [18]. Regardless of whether sPMCA reactions mixtures consisting of purified hamster PrP^C^ and polyA were seeded with hamster strains Sc237 or 139H or conducted as non-seeded reactions, the newly produced PrP^Sc^ gave rise to the same disease phenotype in hamsters [18].

What are the minimal molecular requirements for a faithful replication of a prion strain in vitro? Can faithful replication of a prion strain be achieved using rPrP that lacks posttranslational modifications? What is the minimal set of cofactors sufficient for a faithful replication of a prion strain in vitro? Do prions from different species rely on different sets of cofactors? The current study reports that faithful replication of hamster strain SSLOW could be achieved in vitro using rPrP as a substrate. We found that a mixture of PE and polyA was sufficient for stable replication of hamster brain-derived SSLOW PrP^Sc^ in sPMCA that use hamster rPrP as a substrate. The disease phenotype generated in hamsters upon transmission of recombinant PrP^Sc^ produced in vitro was strikingly similar to the original SSLOW diseases phenotype with respect to the incubation time to disease, clinical, neuropathological, biochemical and structural features of PrP^Sc^, as indicated by infrared microspectroscopy. The current study is the first to demonstrate that rPrP can support replication of brain-derived PrP^Sc^ while preserving its strain identity.

## Materials and methods

### Brain material

Hyper and Drowsy scrapie brain materials were kindly provided by Richard Bessen (Colorado State University, Fort Collins, CO); 263K was kindly provided by Robert Rohwer (Veterans Affair Maryland Health Care System, Baltimore, MD); one 263K scrapie hamster brain used for preparation and FT-IR analysis of highly purified PrP^Sc^ was taken from the prion tissue archive at the Robert Koch-Institute; SSLOW scrapie brain homogenate was prepared using animals from the 4th passage of SSLOW [45]; atypical PrPres was generated from brain material in vitro as described [49]. Ten percent (wt/vol) brain homogenates (10% BH) were prepared in PBS, pH 7.4, using glass/Teflon homogenizers attached to a cordless 12 V compact drill (Ryobi) as previously described [45]. To seed PMCA, 10% BH was diluted in PMCA conversion buffer [44] and briefly sonicated immediately before use.

### Expression and purification of rPrP

Syrian hamster full-length rPrP encompassing residues 23–231 was expressed and purified according to a previously described procedure [9] with minor modifications [43]. Immediately before use, lyophilized rPrP was dissolved in 10 mM Na acetate, pH 5.0, filtered through 0.45 μm syringe filter and the rPrP concentration was measured. For the formation of rPrPres^PolyA^, lyophilized rPrP was dissolved in 5 mM MES, pH 6.0.

### Cofactors

L-α-phosphatidylethanolamine (PE) from porcine brain (#840022C, Avanti Polar Lipids, Alabaster, AL) was supplied as 25 mg/ml stock in chloroform. Immediately before use, an aliquot of PE was lyophilized in a glass tube under a stream of compressed nitrogen, and then re-suspended at 10 mM in 0.05% Triton-X by sonication in a water bath until clear. PolyA (#P9403, Sigma) was dissolved at 5 mg/ml in 10 mM Tris, pH 8.0, 1 mM EDTA buffer, and stored frozen in aliquots.

### Protein misfolding cyclic amplification (PMCA)

QSonica S-3000, S-4000 or Q700 sonicators (Newtown, CT) equipped with a microplate horn were used for PMCA reactions. Samples in 0.2 ml thin-wall PCR tubes (Fisher #14230205) were placed in a floating rack inside the horn filled with 300 ml water and covered with foil. To maintain 37 °C temperature, two coils of rubber tubing attached to a circulating water bath were installed inside the horn. Alternatively, the horn and its enclosure were put inside 37 °C incubator.

rPrPres^PE^ was produced in 20 mM Tris, pH 7.5, 135 mM NaCl, 5 mM EDTA and 0.15% Triton X-100 supplemented with 5 μg/ml rPrP and 2.5 mM PE. For the first round of PMCA, 90 μl aliquots of reaction mixture were supplemented with 10 μl of diluted scrapie brain homogenates as indicated (see Brain Material). PMCA sonication program consisted of 5 s sonication pulses at 150–200 W applied every 10 min during a 24 h period. For each subsequent round, 30 μl of the reaction from the previous round were added to 60 μl of fresh substrate. Each PMCA reaction was carried out in the presence of two 3/32” Teflon beads (McMaster-Carr). To analyze production of PK-resistant PrP material in PMCA, the samples were digested with 10 μg/ml PK at 37 °C for 1 h. The digestion was terminated by addition of SDS-sample buffer and heating the samples for 10 min in a boiling water bath.

rPrPres^PE + PolyA^ was produced in the presence of 2.5 mM PE and 20 μg/ml polyA using the same conditions as for the reactions with PE as a sole cofactor, except the Teflon beads were omitted, and for each subsequent round, 20 μl of the reaction mixture from the previous rounds were added to 80 μl of fresh substrate. The ingredients were mixed in the following order. A master mix consistent of water, Tris, NaCl, EDTA and Triton X-100 was prepared first. Then rPrP was mixed with PE and incubated for 10 min at room temperature. After that, the polyA was added, followed by additional 5 min incubation. Finally, the master mix and rPrP mix were combined and aliquoted. The 90 μl aliquots for the first round were supplemented with 10 μl of seeds and subjected to sonication immediately. The 80 μl aliquots for the subsequent rounds were frozen at − 20 °C. Completed rounds were used to seed the following rounds on the same day. The leftovers were kept frozen before the subsequent analysis. PK-digestion was performed with 10 μg/ml PK at 37 °C for 1 h and terminated by addition of SDS-sample buffer and heating the samples for 10 min in a boiling water bath.

To establish amplification efficiency in a standard PMCA, 10% normal brain homogenate (NBH) from healthy hamsters was prepared as described previously [44] and used as a substrate [26]. To produce desialylated substrate (dsNBH), 10% NBH was treated with *Arthrobacter ureafaciens* sialidase (cat # N3786, Sigma-Aldrich, St. Louis, MO) as described before [33]. For the first round, 90 μl of NBH or dsNBH were supplemented with 10 μl of scrapie brain homogenates serially diluted in PBS. The standard sonication program consisted of 20 s sonication pulses at 150–200 W applied every 20 min during a 24 h period. For each subsequent round, 10 μl of the reaction mixtures from the previous round were added to 90 μl of fresh substrate. Each PMCA reaction was carried out in the presence of two 3/32” Teflon beads (McMaster-Carr). To analyze production of PK-resistant PrP material in PMCA, 10 μl of sample were supplemented with 5 μl of SDS and 5 μl of PK to a final concentration of SDS and PK of 0.25% and 50 μg/ml, respectively, followed by incubation at 37 °C for 1 h. The digestion was terminated by addition of SDS-sample buffer and heating the samples for 10 min in a boiling water bath.

Formation of PrPres^PolyA^ was achieved by serial PMCA of 5 μg/ml rPrP in the buffer conditions similar to previously described [18]: 20 mM MOPS, pH 7.5, 150 mM NaCl, 500 mM Imidasole, 0.5% Triton X-100, 50 mM EDTA with the addition of 20 μg/ml PolyA. No seeds were added to these reactions. PMCA sonication program consisted of 30 s sonication pulses at 150–200 W applied every 30 min during a 24 h period. For each subsequent round, 10 μl of the reaction from the previous round were added to 90 μl of fresh substrate. To analyze production of PK-resistant PrP material in PMCA, the samples were digested with 20 μg/ml PK at 37 °C for 1 h. For PK-digestion pattern comparison, R-fibrils (F^2M^) produced from rPrP as described previously [41] were digested with 1:5000 PK:rPrP ratio at 37 °C for 1 h. The digestion of all samples was terminated by addition of SDS-sample buffer and heating the samples for 10 min in a boiling water bath.

### Bioassay

Each animal received 50 μl (S. hamsters) or 20 ul (Tg7 mice) of inoculum intracerebrally, under 2% O_2_/4 minimum alveolar concentration (MAC) isoflurane anesthesia. After inoculation, animals were observed daily for disease using a ‘blind’ scoring protocol. Non-habituating startle response to sound and/or touch was considered the first clinical sign and was marked as an onset of the disease when consistently observed during consecutive scoring sessions. The animals were euthanized at a terminal stage when unable to rear and having troubles to right themselves after flipping to their backs.

### PrP^Sc^ detection by Western blot

An aliquot of 10% BH was mixed with an equal volume of 4% sarcosyl in PBS, supplemented with 50 mM Tris, pH 7.5, and digested with 20 μg/ml PK (New England BioLabs) for 30 min at 37 °C with 1000 rpm shaking using a DELFIA plate shaker (Wallac) placed in 37 °C incubator. PK digestion was stopped by adding SDS sample buffer and heating the samples for 10 min in a boiling water bath. Samples were loaded onto NuPAGE 12% Bis-Tris gels, transferred to PVDF membrane, and probed with 3F4 or SAF-84 antibody [45].

### Analysis of conformational stability and proteinase K resistance

Ten percent brain homogenate was diluted 10 times into PMCA conversion buffer, then supplemented with an equal volume of GdnHCl solution in PBS to a final concentration of GdnHCl ranging from 0.4 to 4 M and incubated at room temperature for 1 h. Next, nine volumes of 2% sarkosyl in PBS were added to all samples followed by 1 h incubation at room temperature, and then the samples were treated with 20 μg/mL PK for 1 h at 37 °C with shaking. The digestion was stopped with 2 mM PMSF, and the proteins were precipitated in four volumes of ice-cold acetone, incubated overnight at − 20 °C, and subsequently centrifuged for 30 min at 16000 x g. Pellets were dried for 30 min, resuspended in 1 × SDS-sample buffer, loaded into NuPAGE 12% bisTris gels, then transferred to PVDF membrane, and stained with 3F4 antibody.

### Histopathological study

Histopathological studies were performed on three animals per group. Formalin fixed brain halves were divided at the midline. Right hemisphere was frozen, and left hemisphere was fixed in 10% neutral buffered formalin solution. Brains were treated for 1 h with 96% formic acid prior to embedding in paraffin to deactivate prion infectivity. Paraffin embedded brains were sliced into 4 μm sections and processed for hematoxylin-eosin stain as well as for immunohistochemistry for PrP using the mouse monoclonal anti-PrP antibody 3F4 (1:1000, Covance, Berkeley, CA, USA), or rabbit anti-glial fibrillary acidic protein (GFAP; 1:500, Novus, Littleton, CO, USA), or rabbit anti-ionized calcium-binding adapter molecule 1 (Iba1; 1:500, Wako, Richmond, VA, USA). Horse radish peroxidase-labeled goat anti-mouse and anti-rabbit antibody (KPL, Milford, MA) were used as secondary antibody. For detection of disease-associated PrP, we applied a pretreatment of 30 min hydrated autoclaving at 121 °C followed by 5 min in 96% formic acid. Detection was performed using DAB Quanto chromogen and substrate (VWR, Radnor, PA).

### Procedure for purification of scrapie material

Extraction of PrP^Sc^ (in the form of PrP27–30) from a 263K scrapie hamster and from three hamsters (*i*, *ii*, and *iii*) from the second passage of SSLOW^PE + PolyA^ for FTIR microspectroscopic analysis was performed as described by Daus et al. [16] with the following modifications: hemispheres of mid-saggitally split hamster brains (approximately 0.5 g) were each homogenized in adjusted volumes of homogenization buffer for the preparation of 10% (*w*/*v*) brain tissue homogenates. From each donor animal, two aliquots of 1 mL of 10% (w/v) brain tissue homogenate were subjected to the extraction procedure. This yielded two final pellets of highly purified PrP^Sc^ per donor animal, each corresponding to 0.1 g of brain tissue. For infrared spectroscopic analysis final PrP^Sc^ pellets were washed in double-distilled water as described [16] and resuspended in 10 μL of double-distilled H_2_O. 1 μL aliquots of these PrP^Sc^ suspensions were transferred for drying onto a CaF_2_ window of 1 mm thickness (Korth Kristalle GmbH, Altenförde, Germany).

### Infrared microspectroscopy (IR-MSP)

IR-MSP analysis of highly purified PrP^Sc^ extracts were carried out as previously described [16]. Briefly, mid-IR spectra were acquired in transmission mode using an IFS 28/B FT-IR spectrometer from Bruker (Bruker Optics GmbH, Ettlingen Germany) that was linked to an IRscope II infrared microscope (Bruker). IR microspectra were recorded with a spatial resolution of approximately 80 μm. Nominal spectral resolution was 4 cm^− 1^, and the zero filling factor was 4. For each background and for each sample spectrum, 512 individual interferograms were averaged, zero-filled and apodized using a Blackman-Harris 3-term apodization function. For each examined PrP^Sc^ extract from one 263K scrapie hamster and from hamsters *i*, *ii*, and *iii* infrared spectra were recorded at three different positions in PrP^Sc^ sample spots dried on CaF_2_ windows. Data acquisition and spectral preprocessing was carried out by utilizing Bruker’s instrument software OPUS v. 5.5. Second derivative spectra were obtained by means of a 9-smoothing point Savitzki-Golay derivative filter. Spectra from the 263K scrapie hamster and the three hamsters *i*, *ii*, and *iii* were vector normalized in the wave number region between 1610 and 1700 cm^− 1^.

## Results

### PolyA as a sole cofactor is not sufficient for assisting conversion of hamster rPrP into PrP^Sc^

Previous studies revealed that RNA molecules including synthetic, homopolymeric nucleic acids such as polyA assisted conversion of PrP^C^ into self-propagating, PK-resistant, PrP^Sc^-like states in sPMCA [17, 20]. Moreover, RNAs were found to facilitate conversion of hamster PrP^C^, but not mouse or vole PrP^C^, into PrP^Sc^ [19]. These results emphasized species-specific differences in biochemical environment important for conversion. Taking previous data into consideration, we decided to test whether PolyA is sufficient for assisting conversion of hamster rPrP into authentic PrP^Sc^ in vitro. To answer this question, non-seeded sPMCA reactions that utilized hamster rPrP as a substrate were carried out in the presence or absence of synthetic polyA. In the presence of polyA, PK-resistant products appeared between 3rd and 5th sPMCA round, whereas no products were detected in the reactions conducted in the absence of polyA (Additional file 1: Figure S1A). The PMCA-derived, PK-resistant products (rPrPres^PolyA^) consisted of expected peptide of ~ 16 kDa and two shorter peptides of 10 kDa and 8 kDa, which were all detectable by SAF-84 antibody. Once formed, rPrPres^PolyA^ was able to propagate in sPMCA with rPrP as a substrate, albeit with some variations in yield (Additional file 1: Figure S1A).

For testing whether rPrPres^PolyA^ is infections, Syrian hamsters and transgenic mice that overexpress hamster PrP^C^ on an ablated background (tg7) were inoculated with PMCA-derived rPrPres^PolyA^ material. Hamsters did not develop any clinical signs of the disease and were euthanized at 661 days postinoculation. No PK-resistant material was found in brains of hamsters by Western blots (Additional file 1: Figure S1B). Despite expression of PrP^C^ at a level of 3.5-fold higher than that in a hamster [34], tg7 mice did not develop any clinical signs of the disease for up to 524 days postinoculation and were euthanized. However, Western blot analysis of tg7 mice revealed PK-resistant products that were detectable by SAF-84 and consisted of three bands with molecular weight of 23, 16 and 10 kDa. Such PK-digested pattern suggests that upon inoculation of rPrPres^PolyA^, tg7 mice produced PrPres state different from authentic PrP^Sc^, but similar to the atypical PrPres described in our previous studies [44, 46, 48, 49]. Serial transmission of rPrPres^PolyA^ in tg7 mice displayed dynamics similar to those previously observed for the serial transmission of atypical PrPres [44, 46, 48, 49]. In a 2nd passage, tg7 mice did not develop clinical disease, yet again three PK-resistant bands of 23, 16 and 10 kDa were observed using SAF-84 antibody (Additional file 1: Figure S1B) documenting a self-replicating nature of this state. In addition, small amounts of PrP^Sc^ were detectable by SAF-84 and 3F4 antibodies (Additional file 1: Figure S1B). In summary, animals inoculated with rPrPres^PolyA^ material did not develop clinical disease nor did they produce PrP^Sc^ in a first passage arguing that rPrPres^PolyA^ preparation does not contain authentic PrP^Sc^.

### Both PE and polyA are required for efficient conversion of hamster rPrP into PrP^Sc^ in vitro

**Fig. 1:**
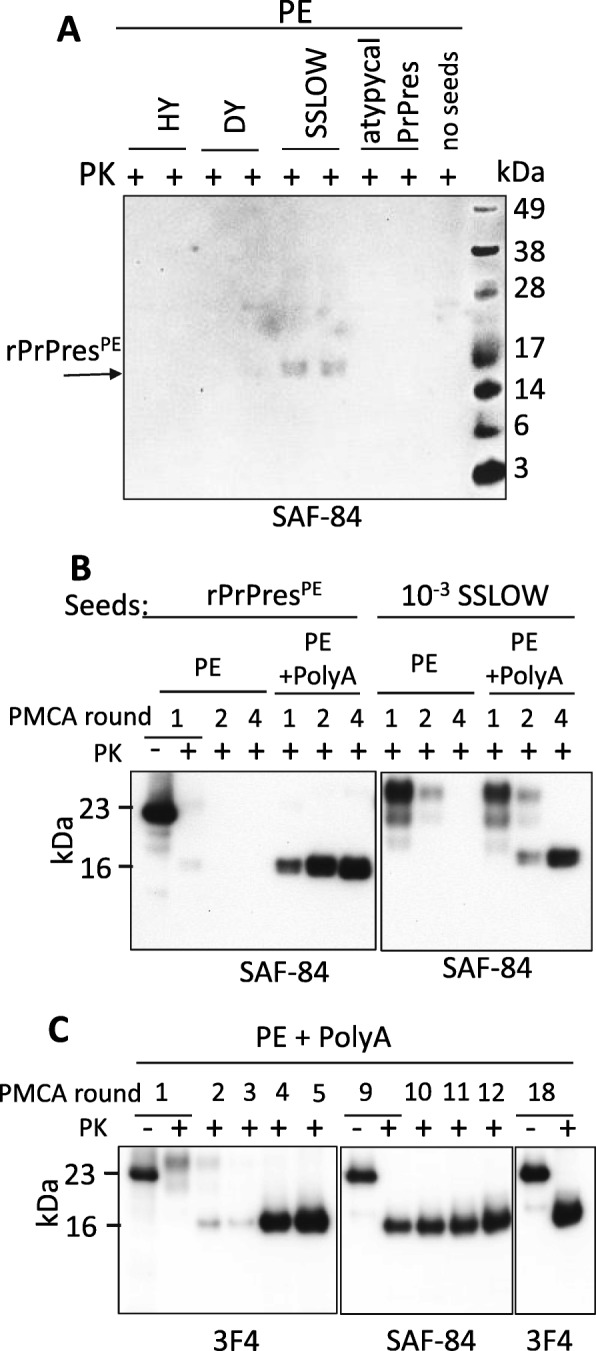
Attempts to produce Ha-rPrP^Sc^ with assistance of PE and PolyA. **a** Duplicate sPMCA reactions were seeded with 10^3^-fold diluted brain-derived Hyper (HY), Drowsy (DY) or SSLOW, or atypical PrPres produced in vitro, then subjected to four sPMCA rounds in the presence of PE with 3-fold dilutions between rounds and analyzed by Western blot. Products of 4th sPMCA rounds are shown. Small amounts of PK-resistant material (rPrPres^PE^) were detected in the reactions seeded with SSLOW (indicated by arrow). **b** sPMCA reactions were seeded with PMCA-derived rPrPres^PE^ or 10^3^-fold diluted SSLOW brain material, then subjected to four serial rounds in the presence of PE alone or a mixture of PE and polyA with 5-fold dilutions between rounds and analyzed by Western blots. **c** Serial amplification of rPrPres^PE + PolyA^ in PMCA. sPMCA reactions were seeded with 10^3^-fold diluted SSLOW brain material, then subjected to 18 serial rounds in the presence of PE and polyA with 5-fold dilutions between rounds and analyzed by Western blots. SAF-84 antibody was used to verify the absence of low molecular weight bands upon PK-digestion (middle panel)

While the experiments on polyA had been carried out, cellular lipids and, specifically, PE were shown to be essential for converting rPrP into infectious PrP^Sc^ in vitro, albeit with a loss of strain identity [21, 22, 63, 64]. Therefore, next we assessed the effect of PE on converting hamster rPrP. sPMCA reactions with rPrP as a substrate were seeded by hamster strains Hyper (HY), Drowsy (DY) or synthetic strain SSLOW. Small amounts of PK-resistant product (referred to as rPrPres^PE^) with molecular weight ~ 16 kDa, expected for recombinant PrP^Sc^, were detected in reactions seeded with SSLOW, but not with HY, DY or non-seeded reactions (Fig. 1a). For testing whether PE also facilitates alternative misfolding pathway leading to atypical PrPres, sPMCA reactions were seeded with brain-derived atypical PrPres, yet no PK-resistant bands were observed (Fig. 1a). Among the strains used for seeding, only reactions seeded with SSLOW showed positive results in the presence of PE (Additional file 1: Figure S2). It is unlikely that this difference could be attributed to the strain-specific differences in the efficiency of amplification in sPMCA, because HY was found to display significantly higher amplification efficiency than SSLOW in conventional sPMCA [24, 27]. Nevertheless, in the presence of PE alone, SSLOW-seeded rPrPres^PE^ propagated with low efficiency and only at low dilution factor between serial PMCA rounds. Next, we tested whether supplementing both polyA and PE will improve the yield and the efficiency of amplification. Serial PMCA reactions were seeded with brain-derived SSLOW or PMCA-derived rPrPres^PE^ and conducted in the presence of a mixture of PE and polyA or PE alone. In both rPrPres^PE^- and SSLOW-seeded reactions, stable amplification of the 16 kDa PK-resistant product was observed only in the presence of a mixture of PE and polyA (will be referred to as rPrPres^PE + PolyA^), but not PE alone (Fig. 1b, c). rPrPres^PE + PolyA^ could be detected by 3F4 antibodies, arguing that the central PrP region that is missing in atypical PrPres was present in rPrPres^PE + PolyA^. No lower molecular weight bands characteristic for atypical PrPres were detected in PK-digested rPrPres^PE + PolyA^ upon immunoblotting with SAF-84 antibody, suggesting that rPrPres^PE + PolyA^ conformation is different from rPrPres^PolyA^.

### rPrPres^PE + PolyA^ is transmissible

**Fig. 2:**
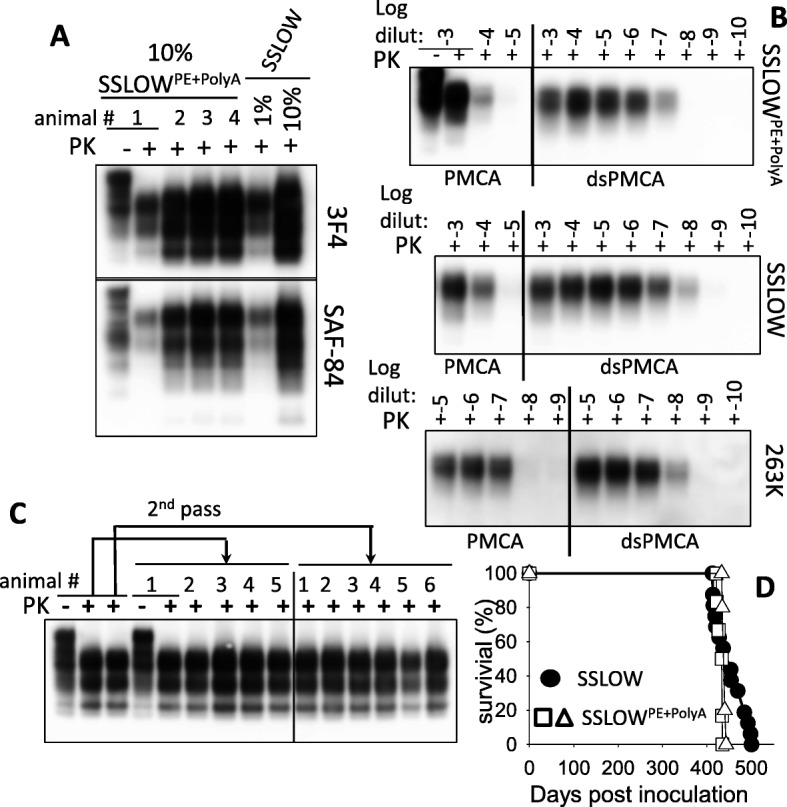
Serial transmission and characterization of the strain produced upon inoculation of rPrPres^PE + PolyA^. **a** Western blots of brain materials from animals inoculated with PMCA-derived rPrPres^PE + PolyA^ and sacrificed at 622 days post-inoculation. One and ten percent brain homogenates from SSLOW-inoculated animals are provided as a reference. **b** Comparison of amplification efficiency of PrP^Sc^ in PMCA and dsPMCA that utilizes desialylated substrate. PMCA or dsPMCA reactions were seeded with SSLOW^PE + PolyA^, SSLOW or 263K brain materials serially diluted to up to 10^10^-fold as indicated, subjected to one amplification round and analyzed by Western blot. In a manner similar to SSLOW, amplification efficiency of SSLOW^PE + PolyA^ increased drastically in dsPMCA conditions relative to that of PMCA conditions. **c** Western blots of brain materials of the first and second passages of SSLOW^PE + PolyA^. Arrows indicate animals that were used for the serial transmission. 3F4 antibody was used. **d** Kaplan-Meier survival curves for animals inoculated with SSLOW (circles) or SSLOW^PE + PolyA^ (2nd passage, two individual groups, squares and triangles)

**Fig. 3:**
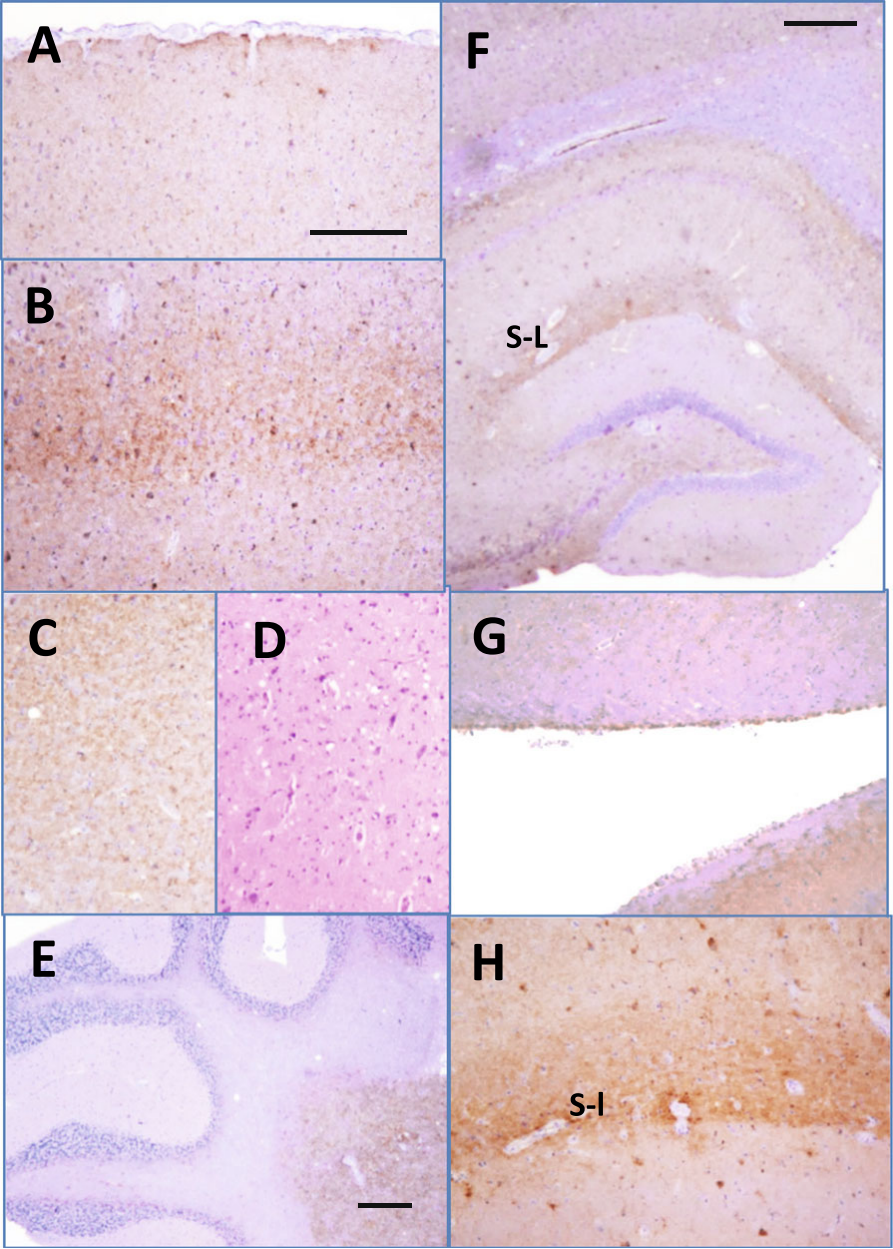
Histopathological analysis of animals from the first passage of rPrPres^PE + PolyA^. PrP^Sc^ was found in form of diffused synaptic deposition and small aggregates in subpial areas (**a**), deep layers of cortex (**b**), thalamus (**c**), cerebellum (**e**), hippocampus (**f**), and subventricular regions (**g**). Stratum-lacunosum (s-l) of hippocampus has characteristically intense deposition of PrP^Sc^ (**f, h**) with a pattern reminiscent of SSLOW [30, 43, 45]. Hematoxylin and eosine staining revealed moderate vacuolation in several brain areas including thalamus (**d**). Scale bars = 200 μm (**a-d**, **g**, **h**) or 300 μm (**e**, **f**)

To test whether rPrPres^PE + PolyA^ is infectious, sPMCA reaction with hamster rPrP was seeded by 10^3^-diluted SSLOW brain material, subjected to 18 rounds of sPMCA (Fig. 1c), then ten-fold diluted sPMCA-derived products were inoculated in Syrian hamsters. The final inoculum contained 1.3 × 10^16^-fold diluted SSLOW brain material; this dilution is approximately 10^7^-fold higher than the limiting dilution of SSLOW as determined by bioassay [47]. Three out of four hamsters inoculated with rPrPres^PE + PolyA^ developed first clinical signs of disease by 517 days postinoculation showing non-habituating startle response to sound and an agitated, fidgeting behavior. With disease progression, three animals with clinical signs showed increasing difficulty righting themselves when rolled onto their back. Their hair became dry and detached in clumps. Three clinical animals were euthanized along with one non-clinical animal at 622 days postinoculation (Table 1). All four animals showed PrP^Sc^ on Western blots detectable by 3F4 and SAF-84 with the lowest amount observed in the sub-clinical animal (Fig. 2a). In three clinical animals the amounts of PrP^Sc^ were similar to those in terminal SSLOW-inoculated animals (Fig. 2a). Histopathological analysis of sick animals revealed accumulation of PrP^Sc^ in forms of diffused synaptic deposits or small aggregates in multiple brain regions including subpial areas, deep layers of cortex, thalamus, hippocampus, cerebellum and subventricular regions (Fig. 3). Notably, hippocampus showed intense deposition of PrP^Sc^ in the stratum-lacunosum region (Fig. 3f, h), a pattern of deposition reminiscent of SSLOW [30, 43, 45]. Hematoxylin and eosine staining revealed moderate vacuolation in several brain areas including thalamus (Fig. 3d). PrP^Sc^ material from animals inoculated with rPrPres^PE + PolyA^ will be referred to as SSLOW^PE + PolyA^.

**Table 1:** Serial transmission of rPrPres^PE + PolyA^ in Golden Syrian hamsters

Passage	n_sick_/n_t_^a^	n _PrP_^Sc^/n_t_^b^	inc time ^c^, dpi	duration ^d^, days
1st	3/4	4/4	517	105
2nd ^e^	6/6	6/6	274, 4 at 300, 324	110, 122, 126, 133, 134, 160
2nd ^e^	5/5	5/5	274, 2 at 288, 324, 335	105, 116, 119, 146, 166

^a^ The number of animals that developed clinical signs per total number of animals

^b^ The number of animals with PrP^Sc^ on Western blot per total number of animals

^c^ Incubation time to first clinical signs

^d^ Duration of clinical disease from the first clinical signs to the terminal stage

^e^ For the second passage, two inocula were prepared using two individual animals from the first passage

For examining strain-specific biochemical properties of SSLOW^PE + PolyA^, we analyzed amplification efficiency of brain-derived PrP^Sc^ using normal PMCA and dsPMCA, in which desialylated substrate is used. Our previous studies revealed that desialylation of PrP^C^ increases the amplification rate in a strain-specific manner. For instance, in dsPMCA the amplification rate of SSLOW PrP^Sc^ increased by several orders of magnitudes relative to its amplification rate in PMCA [33]. PMCA and dsPMCA reactions were seeded with serially-diluted brain-derived SSLOW^PE + PolyA^, SSLOW or 263K and amplified in one round. The amplification rate of 263K increased only by 10-fold in dsPMCA relative to that of PMCA, whereas the amplification rates of SSLOW^PE + PolyA^ and SSLOW increased by approximately 10^3^–10^4^ folds. This experiment illustrates that SSLOW^PE + PolyA^ material has amplification dynamics similar to that of SSLOW.

**Fig. 4:**
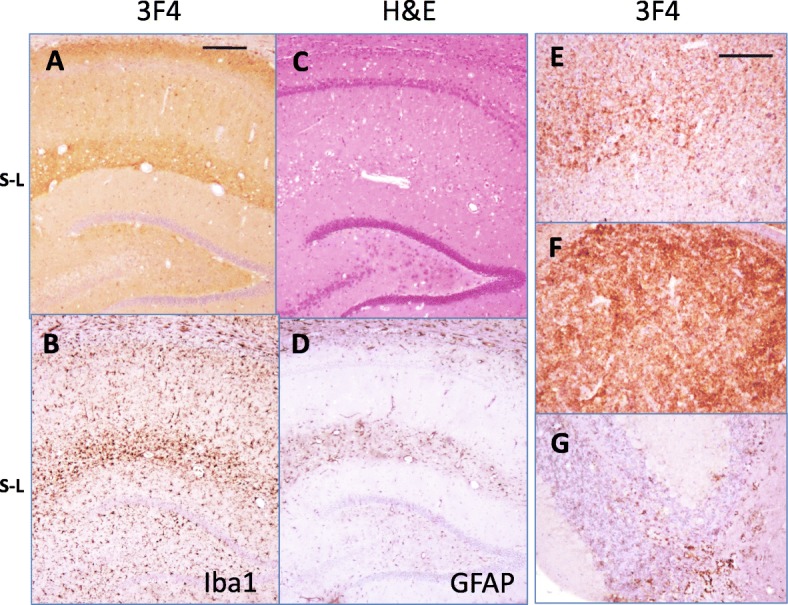
Histopathological analysis of animals from the second passage of rPrPres^PE + PolyA^. PrP^Sc^ deposition in hippocampus (**a**), deep layers of cortex (**e**), thalamus (**f**), and cerebellum (**g**) as shown by 3F4 staining. Vacuolation and reactive gliosis were observed in the areas of PrP^Sc^ accumulation, as shown for hippocampus using staining with hematoxylin and eosine (**c**), or staining of microglia with anti-Iba1 (**b**) or astrocytes with anti-GFAP (**d**). Scale bars = 300 μm (**a-d**) or 200 μm (**e-g**)

For assessing transmissibility of SSLOW^PE + PolyA^, 10% SSLOW^PE + PolyA^ brain homogenates from two animals were inoculated into two new hamster groups. Animals of both groups developed first clinical signs of the disease between 274 and 335 days postinoculation (Table 1). The clinical disease progressed slowly and all animals approached terminal stage by 420–440 days postinoculation (Table 1, Fig. 2c, d). For the second passage of SSLOW^PE + Poly^, the incubation time to the first clinical signs, the duration of the clinical diseases, the timing of terminal stage of the disease and set of clinical signs were highly reminiscent to SSLOW (Fig. 2d) [45]. Non-habituating startle response to sound and touch and an agitated, fidgeting behavior were the first signs of the disease. As the disease progressed the animals had increasing difficulty righting themselves when rolled onto their back. The hair became dry and detached in clumps. Most animals appeared overweight, pear shaped with enlarged abdomens and hind quarters. At the time of euthanasia the animals had become less active and unable to rear. Histopathological examination revealed strong PrP^Sc^ deposition in multiple brain areas including deep layers of cortex, thalamus, hippocampus and cerebellum (Fig. 4a, e, f, g). Similar to SSLOW, SSLOW^PE + Poly^ was characterized by strong perivascular deposition of PrP^Sc^ (Fig. 4g). Vacuolation and neuroinflammation of microglia and astrocytes were observed throughout the brain, but particularly strong in the areas of intense PrP^Sc^ accumulation (Fig. 4b, c, d).

### SSLOW^PE + PolyA^ displays SSLOW-specific structural and neuropathological features

**Fig. 5:**
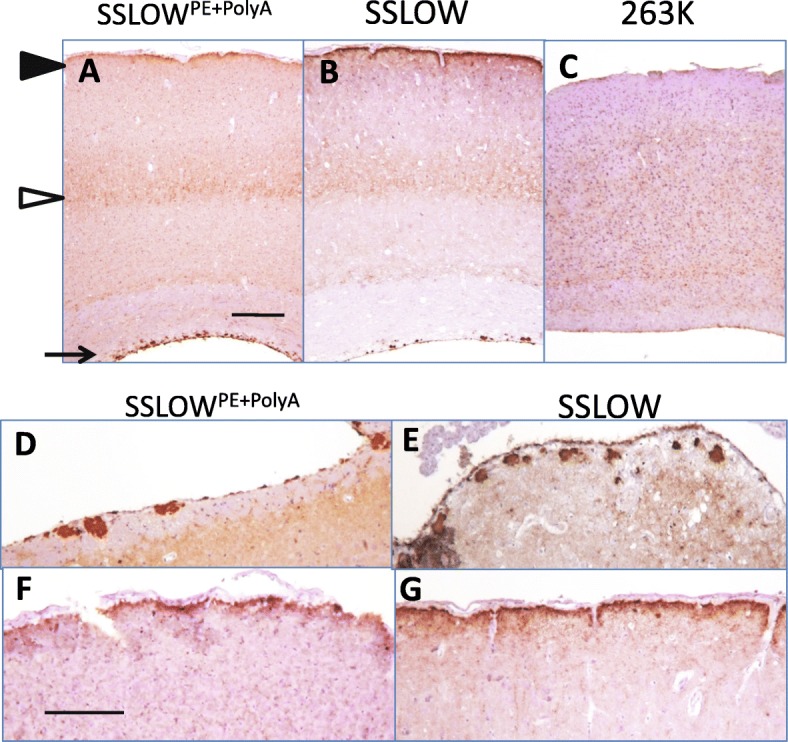
Comparison of neuropathological features of SSLOW^PE + PolyA^ and SSLOW. **a**, **b** Both SSLOW^PE + PolyA^ and SSLOW shows similar patterns of PrP^Sc^ accumulation in cortex including deposition in subpial area (black arrowhead), strong deposition in deeper layers of cortex (white arrowhead), and plaques in subependymal areas (arrow). **c** Cortex of 263K-infected animals displays different pattern of PrP^Sc^ deposition (**d** – **g**). Subependymal plaques (**d**, **e**) and subpial deposition of PrP^Sc^ (**f**, **g**) in SSLOW^PE + PolyA^ (**d**, **f**) and SSLOW (**f**, **g**) animals. Scale bars = 300 μm (**a-c**) or 200 μm (**d-g**)

The question of considerable interest is whether propagation of SSLOW in vitro with an assistance of PE and polyA preserved strain-specific disease phenotype. For addressing this question, first we examined patterns of PrP^Sc^ deposition peculiar to SSLOW. SSLOW is characterized by intense accumulation of diffuse PrP^Sc^ in the stratum lacunosum-moleculare of the hippocampus, which is accompanied by a marked astrocytic inflammation in the same area [30]. Analysis of SSLOW^PE + Poly^ revealed intense deposition of PrP^Sc^ and astrocytic gliosis in stratum lacunosum-moleculare typical for SSLOW (Fig. 4a, d). In a manner similar to SSLOW, SSLOW^PE + Poly^-infected animals also displayed other SSLOW-specific neuropathological features including diffuse PrP^Sc^ in deeper layers of cortex, intense narrow PrP^Sc^ staining in subpial area and PrP^Sc^ plaques in subependymal areas (Fig. 5a-g). Overall, the region-specific intensity of PrP^Sc^ deposition was very similar in animals infected with SSLOW^PE + Poly^ and SSLOW, where thalamus and subependymal areas displaying the strongest deposition, and cerebellum the weakest (Figs. 4 and 5) [43, 45].

**Fig. 6:**
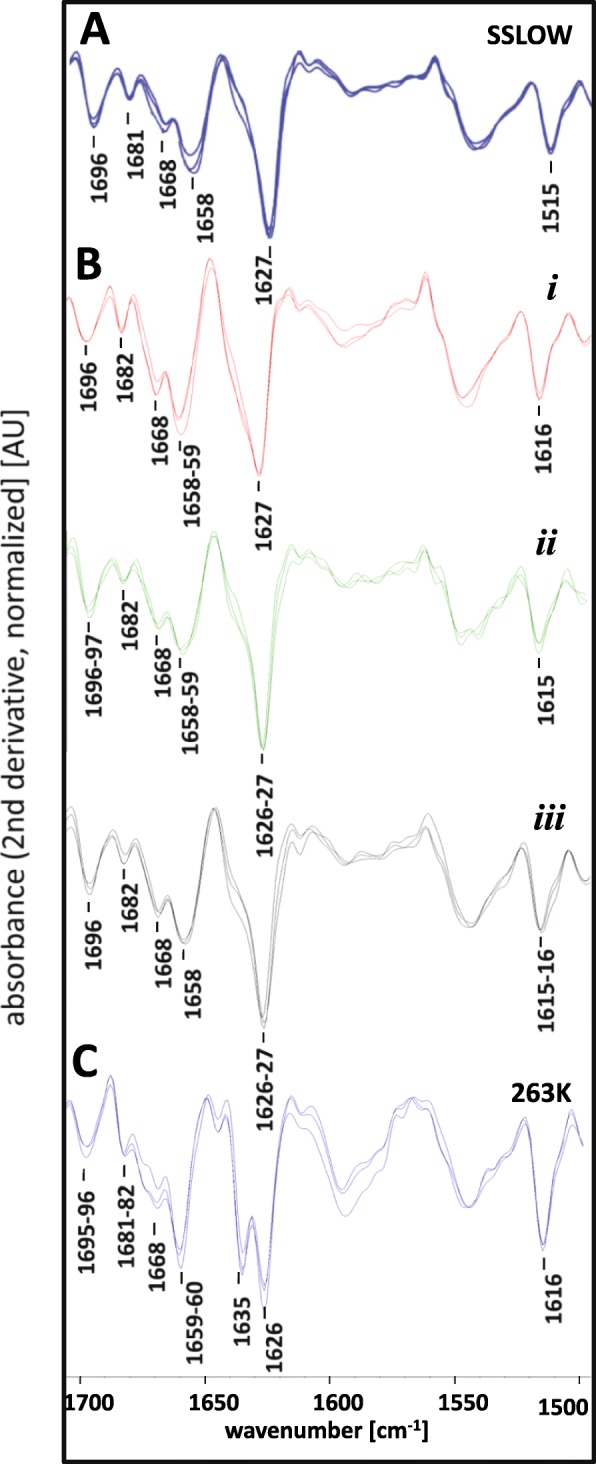
Comparison of the secondary structure of PrP^Sc^ materials by infrared microspectroscopy. **a** Second derivative IR microspectra obtained from PrP^Sc^ material purified in the form of PrP27–30 from three individual SSLOW-infected hamsters (these spectral data originate from the study [31]). **b** Second derivative IR microspectra obtained from PrP^Sc^ material purified in the form of PrP27–30 from three individual hamsters (*i*, *ii*, and *iii*) from the second passage of SSLOW^PE + PolyA^. Technical replicate spectra were acquired for each hamster at three different positions in PrP^Sc^ sample spots dried on CaF_2_ windows. **c** Second derivative IR microspectra obtained from PrP^Sc^ material purified in the form of PrP27–30 from one 263K scrapie hamster brain. Technical replicate spectra were acquired at three different positions in PrP^Sc^ sample spots dried on a CaF_2_ window*.* Similar spectra of highly purified PrP^Sc^ from 263K scrapie hamster brain were previously published elsewhere [16, 57]

Second, PrP^Sc^ was purified in the form of PrP27–30 from SSLOW^PE + Poly^-infected animals and its structural features were examined using infrared microspectroscopy (Fig. 6). Infrared spectroscopy and microspectroscopy have been established as a powerful analytical tool for the detection of structural differences between PrP^Sc^ from various native prion strains or PMCA parent and progeny seeds [13, 16, 31, 55, 61]. SSLOW^PE + Poly^ and SSLOW showed very similar if not identical IR spectra that were characterized by a major peak at 1626–1627 cm^− 1^ that indicates the presence of β-sheet secondary structure elements, a small peak at 1696 cm^− 1^ that also reports on β-sheet structures and a moderate peak at 1658–1659 cm^− 1^ which is typically assigned to an α-helical conformation and/or disordered structures [3, 4] (Fig. 6a, b). The shape of IR spectra displayed by SSLOW^PE + Poly^ and SSLOW was markedly different from that of 263K (Fig. 6c) [16, 57].

Third, we examined conformational stability of PrP^Sc^ in GdnHCl-induced denaturation experiment. The conformational stability was found to be similar for four groups examined: SSLOW^PE + Poly^ from the 1st and 2nd passages, SSLOW and 263K (Additional file 1: Figure S3A). This result is consistent with the previous study that documented similar stability of SSLOW and 263K [43]. However, the band pattern was different between 263K and the three members of SSLOW group (SSLOW^PE + Poly^ from the 1st and 2nd passages, SSLOW) (Additional file 1: Figure S3A). Because electrophoretic mobility of PK is similar to the band corresponding to diglycosylated PrP isoform, the area on the membrane where PK is present has reduced binding of anti-prion antibody, which creates a blind spot. Since PK-related blind sport serves as an internal reference for each lane of SDS-PAGE, this approach was found to be reliable for comparing minor differences between electrophoretic mobility of prion strains, as documented in our previous studies [32]. We found that the PK-related blind spot hides the upper half of diglycosylated PrP band for SSLOW group, while it cuts though the middle of the band for 263K, illustrating slightly higher electrophoretic mobility of the members of SSLOW group relative to that of 263K (Additional file 1: Figure S3A). Direct comparison of electrophoretic mobility confirmed that SSLOW^PE + Poly^ and SSLOW displayed slightly higher mobility in comparison to 263K (Additional file 1: Figure S3B).

## Discussion

In the recent decade, enormous progress has been made in generating highly infectious prions in vitro using rPrP as a substrate. Several experimental protocols for converting rPrP into PrP^Sc^ have been developed that highlight importance of lipids and/or polyanionic molecules for assisting rPrP conversion in vitro [21–23, 35, 63, 67]. These studies established that rPrP that lacks posttranslational modification is able to support replication of highly infectious PrP^Sc^, yet it remains unclear whether prion replication in rPrP can preserve strain identity. In previous studies, seeding of rPrP by brain-derived PrP^Sc^ gave rise to new prion strains with new disease phenotypes documenting loss of a strain identity upon replication in rPrP substrate [21–23, 35]. Remarkably, loss of prion strain identity upon replication in rPrP was mirrored by the studies conducted in transgenic mice with deficient posttranslational modifications of PrP^C^ [1, 40]. Transmission of mouse-adapted prion strain RML or mCWD to transgenic mice expressing PrP^C^ devoid of GPI anchor and deficient in N-linked glycosylation led to formation of novel prion strains, which maintained their novel properties upon transmission to wild type mice [1, 40]. In a similar fashion, passaging of prion strains through transgenic mice expressing PrP^C^ devoid of just N-linked glycans resulted in changes of strain-specific infectious properties upon passaging back to wild type host [11].

In the current study, replication of hamster strain SSLOW was achieved in vitro using rPrP as a substrate with assistance of the mixture of polyA and PE. The disease phenotype generated upon transmission of rPrPres^PE + PolyA^ in hamsters was strikingly similar to the original SSLOW diseases phenotype. In the second passage of SSLOW^PE + Poly^, the incubation time to the first clinical signs, the duration of the disease progression, the incubation time to terminal stage and the set of clinical signs were highly reminiscent to the characteristics of prion infection by SSLOW (Fig. 2d). Moreover, neuropathological analysis demonstrated remarkable resemblance between animals affected by SSLOW^PE + Poly^ and those affected by SSLOW with respect to brain regions affected by PrP^Sc^ and PrP^Sc^ deposition patterns (Figs. 4 and 5). Analysis of purified PrP^Sc^ using infrared microspectroscopy indicated that SSLOW^PE + PolyA^ and SSLOW had very similar, if not identical, secondary structures (Fig. 6). IR-MSP is very sensitive technique which can be used to detect even very small structural changes [13, 16, 31, 55, 61]. While the fact that we cannot detect any spectroscopic differences does not fundamentally exclude conformational differences, yet our IR-MSP results indicate that conformational differences, if there are any, must be very small. In addition to similar secondary structures, SSLOW^PE + PolyA^ and SSLOW PrP^Sc^ showed very similar amplification dynamics in PMCAs that employed normal and desialylated substrate (Fig. 2b). Finally, SSLOW^PE + PolyA^ and SSLOW PrP^Sc^ displayed similar electrophoretic mobility, which was slightly faster in comparison to that of 263K (Additional file 1: Figure S3). The current study is the first to demonstrate the proof of principle that rPrP is capable of preserving strain identity of brain-derived PrP^Sc^.

In previous studies, the majority of work on generating infectious recombinant prions has been conducted using mouse rPrP [21, 22, 63, 67]. The current study is the first to document that successful propagation of a hamster strain could be achieved in vitro using hamster rPrP. Propagating of hamster strains in vitro using rPrP or unglycosylated PrP^C^ was found to be very challenging. All hamster strains, whether of natural or synthetic origin, are predominantly diglycosylated [2, 27]. In fact, previous studies showed that diglycosylated PrP^C^ molecules were required for propagating hamster Sc237 strain in PMCA [50]. Surprisingly, while unglycosylated mouse PrP^C^ were required for replicating mouse prions, unglycosylated hamster PrP^C^ molecules inhibited replication of hamster prions [50]. In vivo, N-linked glycans might play a role in facilitating the assembly of hamster PrP^Sc^ or stabilizing PrP molecules within hamster PrP^Sc^ [50]. The current work provides a proof of principle that faithful replication of hamster prion strain that typically relies on diglycosylated PrP^C^ molecules could be achieved in the absence of N-linked glycan, but with assistance of two cofactors.

It is not clear whether the results presented in the current study represent a rare exception or general rule. We do not know whether other hamster-adapted strains might have more stringent requirements for propagation using rPrP as a substrate including not only a different set of cofactors, but also PMCA amplification conditions (dilution between rounds, sonication time and power). While failure of DY to utilize rPrP substrate in the current study could be attributed to its very low rate of replication, as assessed in conventional PMCA reactions [2], this is not the case for HY. In fact, with PrP^C^ as a substrate the replication rate of HY was found to be faster than that of SSLOW [27]. One possibility behind faithful replication of SSLOW in rPrP substrate could be attributed to its synthetic origin, as it was generated via serial transmission of rPrP amyloid fibril prepared in vitro [43]. However, such possibility, should be considered with great caution, because structure of rPrP fibrils that gave rise to SSLOW were fundamentally different from that of authentic PrP^Sc^ including SSLOW PrP^Sc^, which emerged in hamster upon serial passaging [51, 66]. In fact, four serial passages in hamsters were required to stabilize SSLOW-specific disease phenotype and PrP^Sc^ properties [42, 45, 48, 49]. While SSLOW is a strain of synthetic origin, animals infected with SSLOW display all key neuropathological and biochemical characteristics of transmissible spongiform encephalopathies including chronic neuroinflammation and neurodegeneration, spongiform vacuolation, deposition of bona fide PrP^Sc^, efficient transmission of the disease between animals, high infectivity titer and efficient amplification of SSLOW PrP^Sc^ in PMCA [30, 38, 45, 47]. In a manner similar to PMCA amplification of hamster strains of natural origin, amplification of SSLOW in PMCA displayed dependence on RNAs, the sialylation status of N-linked glycans, and also showed a species barrier upon amplification in mouse substrate [26, 27, 33].

Previous studies documented that RNA molecules including polyA facilitate in vitro replication of hamster prion strains in PMCAs that employ brain-derived hamster PrP^C^ as a substrate [18, 20]. Notably, the effect of RNAs on stimulating prion replication was found to be species- and strain-dependent [19, 27, 54]. RNAs had strong stimulating effects on replication of hamster strains, yet their effect on mouse strains was considerably less pronounced and strain-dependent [19, 27, 54]. The current study demonstrated that polyA can facilitate misfolding of hamster rPrP (Additional file 1: Figure S1). However, in the presence of polyA as a sole cofactor, rPrP misfolded into PK-resistant, self-replicating state, which was different from PrP^Sc^ (Additional file 1: Figure S1). These data suggest that polyA and, perhaps, other RNAs indeed promote PrP misfolding, however without imposing strict constraints with respect to misfolding pathways. Interestingly, our previous studies demonstrated that serial replication of hamster strain 263K in PMCA conducted in RNA-depleted brain homogenates resulted in self-replicating PrP states that failed to produce prion disease in hamsters [25, 32] .

In previous studies, PE was found to be sufficient as a sole cofactor for propagating mouse prion strains in vitro and generating highly infectious recombinant PrP^Sc^ from rPrP [21, 22]. In the present work, we were not able to efficiently propagate SSLOW using rPrP as a substrate in the presence of PE as a sole cofactor (Fig. 1b). The current results are consistent with previous observations that replication of hamster strains exhibit stronger dependency on polyanions than mouse strains [19]. Our work also suggests that faithful replication of prion strains from different species using rPrP might requires different sets of cofactors.

The current study demonstrates that rPrP can support replication of brain-derived PrP^Sc^ preserving its stain identity despite lack of posttranslational modifications. In contrast to rPrP, PrP^C^ that serves as a replication substrate in a brain is posttranslationally modified with GPI anchor and N-linked glycans [58, 59, 62]. Previously, we proposed that in PrP^C^, posttranslational modifications might limit the diversity of misfolding pathways that are otherwise accessible to rPrP [6, 10, 36]. Consistent with this view, previous studies documented changes in strain-specific disease phenotype and physical properties of PrP^Sc^ upon passaging of prion stains in transgenic mice expressing PrP^C^ devoid of GPI anchor and/or N-linked glycans [1, 11]. In the absence of posttranslational modifications and cofactors, rPrP alone displays a broad spectrum of misfolding pathways [7, 36, 41]. What is the mechanism behind PE-assisted conversion of rPrP into PrP^Sc^? Our previous studies that employed steady-state spectroscopic techniques failed to find any evidence of direct physical interactions between PE and rPrP [56]. Bearing this in mind, one could propose that interactions between PE and rPrP are very weak and/or transient (PE-rPrP complexes exists for very short time periods). If this is the case, only a tiny fraction of rPrP could be found in a state bound to PE at any given time, the fraction that could be presumably an intermediate toward PrP^Sc^. According to this mechanism, PE could promote misfolding of rPrP directly, along the pathway that leads to PrP^Sc^. Alternatively, PE might assist rPrP conversion into infectious states indirectly, i.e. by binding and neutralizing intermediates toward alternative, non-infectious amyloid states. This mechanism proposes that PE might limit the diversity of misfolding pathways. If this is the case, one would expect that PE would promote replication of other hamster strains, which was not supported by current observations. A third possibility is that PE is involved transiently at the stage of interaction of rPrP with PrP^Sc^ seeds. Whether such transient interactions depend on strain-specific properties of PrP^Sc^ seeds remains to be established. Regardless of the specific mechanism, PE was found to be essential for propagating SSLOW-specific features using rPrP.

Incomplete attack rate and prolonged incubation time to disease observed in the first passage of SSLOW^PE + PolyA^ argues that sPMCA-derived rPrPres^PE + PolyA^ material had low specific prion infectivity (Table 1). A drop in specific prion infectivity could be due to accumulation of alternative, non-infectious, self-replicating states that replicate faster than SSLOW PrP^Sc^ in sPMCA with rPrP. In addition, such drop could also be due to conformational changes and/or changes in size of SSLOW PrP^Sc^ particles during sPMCA. Notably, the diminished specific prion infectivity in sPMCA is not specific to sPMCA that employs rPrP as a substrate, as it was previously documented for conventional sPMCAs conducted with PrP^C^ as a substrate. In fact, previous studies established that replication of hamster strains including 263K and SSLOW in sPMCA reactions consisting of multiple rounds reduced prion infectivity [31, 37]. In our previous study, hamsters inoculated with sPMCA-derived SSLOW subjected to 24 rounds of PMCA in normal brain homogenates did not develop clinical diseases for at least 621 days postinoculation, but showed PrP^Sc^ accumulation in their brains and spleens [31]. Nevertheless, the fact that SSLOW^PE + PolyA^ animals from the 2nd passage showed incubation time to diseases, disease phenotype and structural PrP^Sc^ features typical for SSLOW argued that at least a fraction of sPMCA-derived rPrPres^PE + PolyA^ material preserved authentic properties of SSLOW upon replication in rPrP.

## Conclusions

The current study is the first to demonstrate that faithful replication of a prion strain that preserves strain-specific identity could be achieved in vitrousing recombinant prion protein despite lack of posttranslational modifications. Faithful replication required two cofactors to be present in the reaction mixture with recombinant prion protein: poly A and phosphatidylethanolamine.

## Additional file

Additional file 1: Supplementary Materials. (PDF 937 kb)

## Abbreviations

GPI: Glycosylphosphatidylinositol
IR-MSP: Infrared microspectroscopy
PE: Phosphatidylethanolamine
PrP^C^: Normal, cellular isoform of the prion protein
PrP^Sc^: Infectious, disease-associated, pathogenic form of the prion protein;
rPrP: recombinant prion protein
sPMCA: serial protein misfolding cyclic amplification
