# Correction to: preserving prion strain identity upon replication of prions in vitro using recombinant prion protein

**DOI:** 10.1186/s40478-018-0601-6

**Published:** 2018-09-24

**Authors:** Natallia Makarava, Regina Savtchenko, Peter Lasch, Michael Beekes, Ilia V Baskakov

**Affiliations:** 1 Center for Biomedical Engineering and Technology, University of Maryland School of Medicine, 111 S. Penn St, Baltimore, MD 21201 USA; 2 Department of Anatomy and Neurobiology, University of Maryland School of Medicine, Baltimore, MD USA; 3 Centre for Biological Threats and Special Pathogens, Robert Koch-Institute, 13353 Berlin, Germany

## Correction

Figure 6 of the original publication [1] contained an error in the Wavenumber in panels B and C. The wavenumbers 1616 (Cm-1) in panels B and C should have been 1516 (cm-1). The updated figure has been published in this correction article; the original article has been updated.

**Fig. 6:**
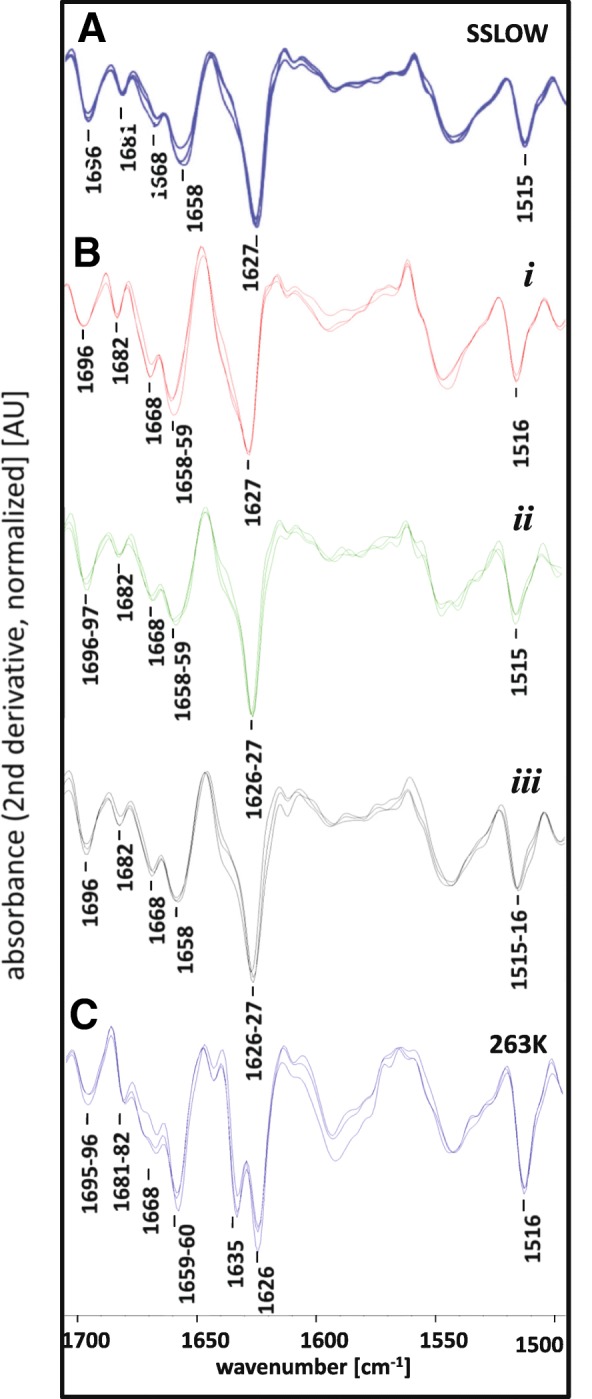
Corrected version of Fig. 6. The wavenumbers in panels B and C have been updated. The wavenumbers in panel A remain the same

## References

[1] Makarava N, Savtchenko R, Lasch P et al (2018) acta neuropathol commun 6(92). 10.1186/s40478-018-0597-y 10.1186/s40478-018-0597-y PMC6134792 30208966

