# Supplementary material for: Preserving prion strain identity upon replication of prions in vitro using recombinant prion protein
Source: Acta Neuropathol Commun. 2018 Sep 12;6:92. doi: 10.1186/s40478-018-0597-y (PMC6134792; doi:10.1186/s40478-018-0597-y)
Supplement: Supplementary file 1 — Supplementary Materials. (PDF 937 kb) [file 40478_2018_597_MOESM1_ESM.pdf]

Supplemental materials

Preserving prion strain identity upon replication of prions *in vitro* using recombinant prion protein

Natallia Makarava[1,2], Regina Savtchenko[1,2], Peter Lasch[3], Michael Beekes[3], Ilia V. Baskakov[1,2]*

[1] Center for Biomedical Engineering and Technology, University of Maryland School of Medicine, Baltimore, Maryland, 21201 United States of America, [2] Department of Anatomy and Neurobiology, University of Maryland School of Medicine, Baltimore, Maryland, United States of America, [3] Centre for Biological Threats and Special Pathogens, Robert Koch-Institute, 13353 Berlin, Germany

Supplemental Figure Legends

Figure S1. An attempt to produce Ha-rPrP^{Sc} with help of PolyA. (A) PolyA facilitates conversion of Ha-rPrP into self-propagating PK-resistant conformation in sPMCAb (left panels show three parallel reactions conducted in the presence of PolyA). As a reference, Ha-rPrP fibrils produced in vitro in the presence of 2 M GdnHCl and digested with PK were loaded onto the gels (F^{2M}). (B) Analysis of Syrian hamster and tg7 mouse brains from animals inoculated with Ha-rPrPres^{PolyA} produced in sPMCAb. Asterisks marks under-digested full-length PrP^{C} and C1 proteolytic fragment. (C) Serial transmission (2nd passage) of Ha-rPrPres^{PolyA} produced in sPMCAb and passaged in Tg7 mice. Small amounts of PrP^{Sc} is detected with SAF-84 and confirmed with 3F4 antibody.

Figure S2. Generation of rPrPres^{PE}. sPMCA reactions were seeded with 10^{3}-fold diluted brain-derived Hyper (HY) or SSLOW, then subjected to four sPMCAb rounds in the presence of PE with 3-fold dilutions between rounds and analyzed by Western blot with 3F4 antibody. rPrPres^{PE} detected in the reactions seeded with SSLOW is indicated by arrow.

Figure S3. Analysis of conformational stability and electrophoretic mobility of SSLOW$^{PE+PolyA}$. (A) 1% BHs from the 1st and 2nd passages of SSLOW$^{PE+PolyA}$, SSLOW and 263K were incubated with increasing concentrations of GdnHCl from 0.4 to 4 M for 1 h, as indicated, then diluted out of GdnHCl, equilibrated for 1 h at room temperature and digested with 20 μg/ml PK. Undigested brain material exposed to 0.4 M GdnHCl is provided as a reference (-PK). Western blots were stained with 3F4 antibody. Dashed arrows point at blind spots on Western blots that arose due to transfer of PK. While all four groups showed similar conformational stability, the band pattern that appeared due to PK-related blind spot was different between 263K and the remaining three groups. (B) 1% BHs from the 2nd passages of SSLOW$^{PE+PolyA}$, SSLOW and

263K were treated with PNGase and PK, and analyzed by Western blot. Western blot was stained with 3F4 antibody.

Figure S1

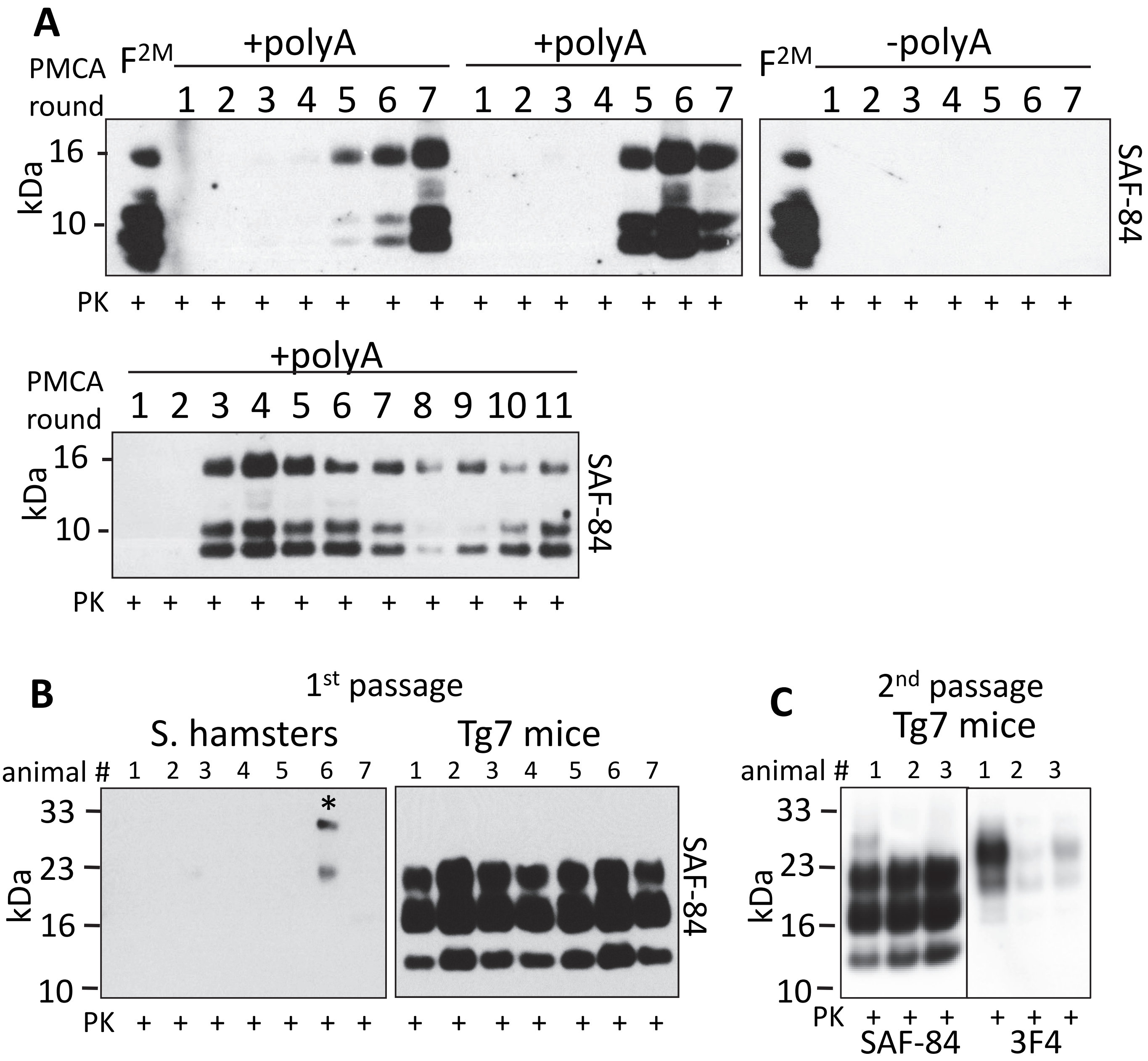
A
PMCA round
F2M
+polyA
+polyA
-polyA
1 2 3 4 5 6 7
kDa
16
10
SAF-84
PK
+polyA
1 2 3 4 5 6 7 8 9 10 11
B
1st passage
S. hamsters
Tg7 mice
animal #
33
23
16
10
*
C
2nd passage
Tg7 mice
SAF-84
3F4

Figure S2

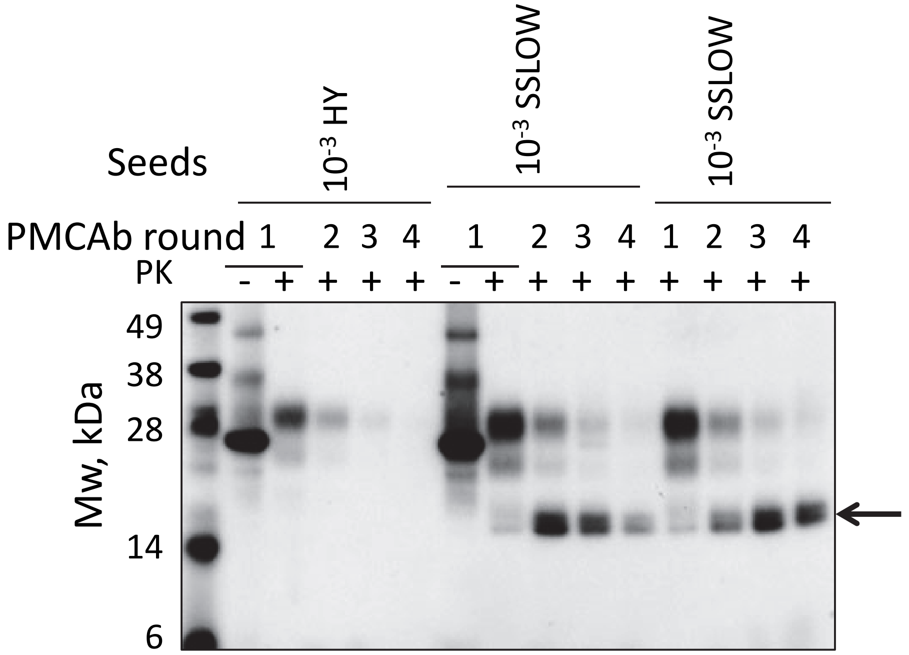

Seeds
10-3 HY
10-3 SSLOW
10-3 SSLOW
PMCAb round 1 2 3 4 1 2 3 4 1 2 3 4
PK - + + + + - + + + + + + + +
49
38
28
14
6
Mw, kDa

Figure S3

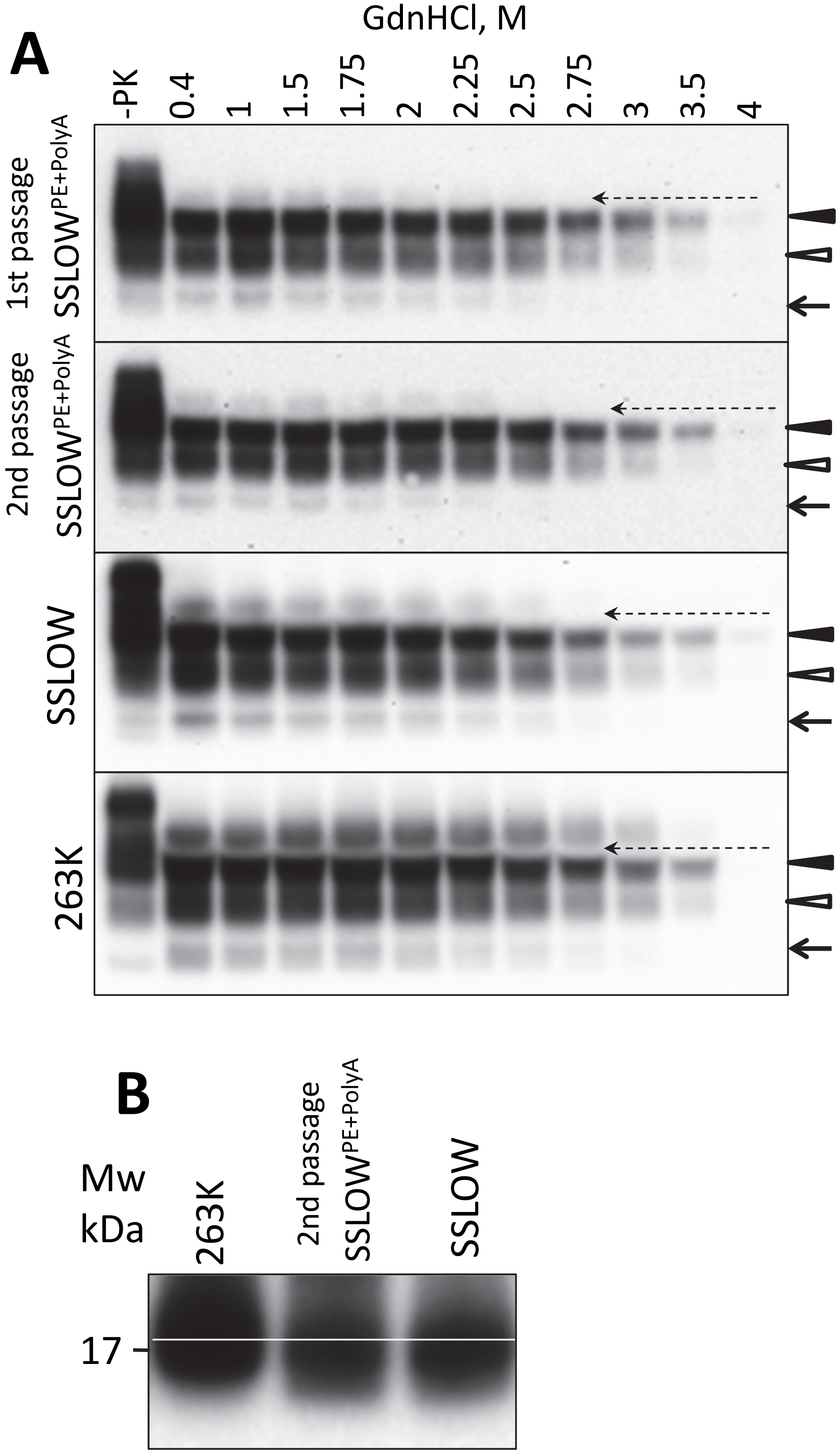
A
GdnHCl, M
-PK
0.4
1
1.5
1.75
2
2.25
2.5
2.75
3
3.5
4
1st passage
SSLOW^PE+PolyA
2nd passage
SSLOW^PE+PolyA
SSLOW
263K
B
Mw
kDa
263K
2nd passage
SSLOW^PE+PolyA
SSLOW
17
